# Extraction of potential adverse drug events from medical case reports

**DOI:** 10.1186/2041-1480-3-15

**Published:** 2012-12-20

**Authors:** Harsha Gurulingappa, Abdul Mateen‐Rajput, Luca Toldo

**Affiliations:** 1, Molecular Connections Pvt. Ltd., Basavanagudi, Bangalore 560004, India; 2, Merck KGaA, Frankfurterstraße 250, Darmstadt 64293, Germany

## Abstract

The sheer amount of information about potential adverse drug events published in medical case reports pose major challenges for drug safety experts to perform timely monitoring. Efficient strategies for identification and extraction of information about potential adverse drug events from free‐text resources are needed to support pharmacovigilance research and pharmaceutical decision making. Therefore, this work focusses on the adaptation of a machine learning‐based system for the identification and extraction of potential adverse drug event relations from MEDLINE case reports. It relies on a high quality corpus that was manually annotated using an ontology‐driven methodology. Qualitative evaluation of the system showed robust results. An experiment with large scale relation extraction from MEDLINE delivered under‐identified potential adverse drug events not reported in drug monographs. Overall, this approach provides a scalable auto‐assistance platform for drug safety professionals to automatically collect potential adverse drug events communicated as free‐text data.

## Background

Adverse drug effects are a very serious issue that confronts patients, healthcare providers, regulatory authorities and drug manufacturers. While stringent measures for detecting risks associated with drug usage are clinical trials, the wide field usage might show additional risks non detectable in the clinical trials due to the limited number of patients involved. After the marketing approval, undesired effect of drugs are reported to the authorities using so called Spontaneous Adverse Event Reporting Systems, that are then timely analyzed to ensure safe use of drugs [1]. A well known problem of pharmacovigilance is however the under reporting, namely the low number of reports that the Authorities receive. Case reports published in the scientific biomedical literature represent an important resource complementary to the SAERS due to their abundant existence, rapid rate of generation, and valuable information enclosed [2]. Due to their unstructured nature, manual analysis of the scientific literature is challenging, cumbersome, and labor intensive. In recent years, development of automatic natural language processing (NLP) and information extraction (IE) techniques have gained large popularity. They include identification of biomedical named entities, relations between the entities, or events associated with them. Noticeable efforts have been invested on mining the potential adverse drug events in different forms of free‐text data. Examples include Wang et. al. [3] who applied the MedLEE system on discharge summaries to identify medication events and entities that could be potential adverse drug effects; these were detected using the strength of statistical association based on their co‐occurrences. Leaman et. al. [4] proposed a lenient NLP model for extracting adverse effects of drugs from social media such as blogs. Gurulingappa et. al. [5] developed a machine learning‐based system for classifying the sentences in MEDLINE case reports that assert potential adverse drug events. However, according to the author’s knowledge, there is a limited focus on identification of semantic relationships between drugs and adverse events in text. This is partly due to the unavailability of suitable open access corpora that could be used for technology development and benchmarking. Extracting relations between drugs and adverse effects can facilitate appropriate indexing, precise searching, visualization, faster information tracing and improve sensitivity of signal detection in pharmacovigilance. The use of ontology of adverse drug events for automated signal generation in pharmacovigilance has already been proposed [6] and its application to information retrieval has been exploited by the same group few years later in the VIGITERMES project [7]. There, the OntoEIM adverse event ontology was used to extend the dictionary of adverse event entities, normalize queries, and consolidate annotations, achieving 29% precision and 67% recall on MEDLINE abstracts. Automatic extraction of potential adverse drug events from clinical records is an active area of research [8]. Mining social internet message boards to identify potential adverse drug events has been reported [9], whereby in that work the extraction of drug‐event pairs was determined only using co‐occurrence of terms within a window of 20 tokens apart, and the use of machine learning systems was only focused on de‐identification for privacy protection. This work reports on the adaptation of a machine learning‐based system for identifying the relations between drugs and adverse effects in MEDLINE case reports; it relies on an ontology‐driven manually annotated corpus that strictly follows semantic annotation guidelines developed for clinical text [10]. The system has been qualitatively evaluated and studied for its ability of support real time pharmacovigilance studies.

## Methods

### Corpus preparation

**Figure 1:**
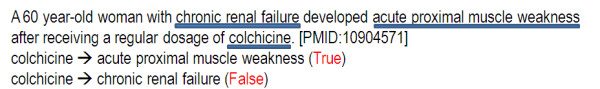
**Example of an annotated sentence in the ADE corpus.** Example of a sentence annotated with drug, conditions, and relations between them in the ADE corpus. *True* indicates presence of adverse effect relation and *False* indicates absence of adverse effect relation.

The data set used for training and validation of the relation extraction system is the ADE corpus [11]. The ADE corpus contains 2972 MEDLINE case reports that are manually annotated in duplicate and harmonized by three annotators. The corpus contains annotations of 5063 drugs, 5776 conditions (e.g. diseases, signs, symptoms), and 6821 relations between drugs and conditions representing clear adverse events. All annotations are confined to sentence level i.e. drugs and conditions representing adverse events co‐occurring only within individual sentences are annotated. Drugs and conditions that are not part of a potential adverse event relation are not annotated. This was done in accordance to the annotation guidelines. The ADE corpus contains annotations of relations between drugs and conditions that represent *True* relations. This represents a sparsely annotated dataset. For training a supervised classifier, it was essential to generate *False* relations i.e. drugs and conditions that do not fall into adverse effect relations but that are still within the same sentence. For this purpose, ProMiner, a dictionary‐based named entity recognition system [12] was employed. ProMiner was incorporated with DrugBank [13] and MedDRA [14] dictionaries for the identification of drugs and conditions respectively in the ADE corpus that were previously not annotated by human annotators. As a result of named entity recognition, new instances encompassing 2269 drugs and 3437 conditions were automatically annotated. Drug‐condition pairs co‐occurring within sentences that were previously not annotated by humans formed *False* relations. Altogether, 5968 *False* relations were automatically generated. The corpus enriched with machine annotated drugs, conditions, and relations between them is referred as ADE‐EXT (indicating extended ADE corpus). Figure 1 shows an illustration of *True* and *False* relations between drug and conditions co‐occurring within a sentence.

In the ADE‐EXT corpus, 120 manually annotated *True* relations were not suitable for the NLP task. Examples include overlapping inter‐related entities such as *acute lithium toxicity* where *lithium* is related to *acute toxicity*. After removal of nested annotations, the ADE‐EXT corpus was decomposed into a training set (ADE‐EXT‐TRAIN) and a test set (ADE‐EXT‐TEST). Counts of entities and relations in subsets of ADE‐EXT corpora are shown in Table 1.

**Table 1:** Counts of entities and relations in ADE‐EXT corpus subsets

**Corpus**	**ADE‐EXT‐TRAIN**	**ADE‐EXT‐TEST**
Documents	1884	210
Drugs	6770	758
Conditions (adverse effect)	8539	978
Sentences	5333	606
*True* Relations	6030	671
*False* Relations	4799	546

### Relation extraction workflow

For the identification and extraction of drug‐condition entity pairs that constitute a potential adverse event relation, the Java Simple Relation Extraction (JSRE) system [15] was employed. JSRE provides a re‐trainable and scalable supervised classification platform that uses Support Vector Machines (SVMs) [16] with different kernels specially designed for the NLP and relation extraction. All sentences in ADE‐EXT‐TRAIN and ADE‐EXT‐TEST containing drug‐condition pairs labelled as either *True* or *False* were transformed into the SRE format before subjecting them to relation extraction. The SRE format is a unique way of representing data within the JSRE platform where tokens appearing in sentences are enriched with their parts‐of‐speech tags, lemmas, and flags indicating if a token is a part of named entity or not. Amongst different kernels available, the shallow linguistic kernel was thoroughly used since it has been widely applied and has shown success during similar relation extraction tasks [17]. The ADE‐EXT‐TRAIN was used as data for training and cross‐validation of JSRE whereas the ADE‐EXT‐TEST was used as an independent test set.

### Mapping annotation ontology against ontology of adverse events

**Figure 2:**
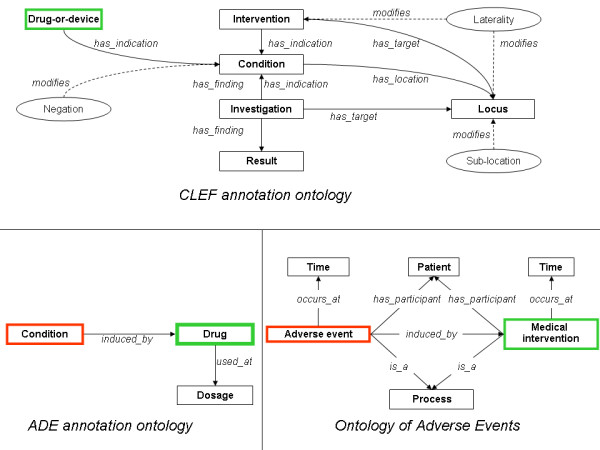
**Ontologies discussed in this work.** Mappings between ADE, OAE, and CLEF ontologies have been shown. Identical entities are in boxes with same colours. *Condition* in the CLEF ontology is mapped to *Process* in the OAE.

The Clinical E‐Science Framework (CLEF) initiative [18] investigated how to generate semantically annotated medical corpora for information extraction. As described by Gurulingappa et. al. [11], we adopted the standard established by the CLEF framework for the annotation workflow [10] however we reshaped the annotation schema by using only two of the original entities (condition, drug) and extended it with a third one (dosage). None of the relationships used by the CLEF annotation schema could be reused for our work, since the CLEF annotation schema did not consider adverse drug events, instead we created two relations: drug‐cause‐condition, drug‐has‐dosage. In this work we focused only on automating the detection of drug‐cause‐condition thus dosage will not be mentioned further. The ADE corpus has been created using the Knowtator plugin for Protégé [19], an ontology‐driven corpus annotation tool also used for the creation of the CLEF corpus. Although we adopted the same tool used in CLEF and also adopted the standard established by the CLEF framework for the annotation workflow, we could not adopt the same annotation ontology since the latter was not able to capture drug‐adverse event and drug‐dosage relations. The annotation ontology described above was therefore used to create the ADE corpus. Subsequent to the corpus creation, the realism‐based biomedical ontology for representation of adverse events (OAE) has been published [20]. OAE has been developed following the principles of Ontological Realism, thus is aligned with the Basic Formal Ontology and the Relation Ontology, and with the Open Biological and Biomedical Ontologies (OBO) Foundry principles of openness, collaboration and use of a common shared syntax. OAE has 484 representational units, annotated by means of 369 terms with specific identifiers and 115 terms imported from existing ontologies. The use of ontologies has proven of great value in biomedicine, also since it enable machine reasoning, abstraction and automatic hypothesis generation. We therefore had interest in investigating if the knowledge encoded in the annotations of the ADE corpus could be semantically connected to the OAE. For doing this, we manually compared the definitions of the entities of OAE and of ADE annotation ontology. Figure 2 shows the basic design patterns of OAE, ADE and CLEF as from the original papers, emphasizing shared entities using green and red colors.

## Results and discussion

### Performance evaluation criteria

The performance of relation extraction was evaluated by 10‐fold cross‐validation of the training data. During cross‐validation of the training data and final evaluation over the test set, classification performances were assessed using the F‐score over *True*‐labelled relations since they represent potential adverse event relations between drugs and conditions that denote a focused relation class being studied.

### Assessment of relation extraction

Baseline experiments began with training and cross‐validation of JSRE over the ADE‐EXT‐TRAIN corpus. Results of system’s performances are shown in Table 2. The system achieved an overall F‐score of 0.87 after cross‐validation. Upon the final test over ADE‐EXT‐TEST, the system attained F‐score of 0.87 indicating a consistency in classification. A subset of instances misclassified during the cross‐validation and testing were manually investigated to understand the common sources of errors. Limited context appeared to be one reason for misclassification. For example, the title *Niacin maculopathy* (PMID:3174043) infers *maculopathy* as a potential adverse event of *niacin* that lacks contextual description to support machine classification. Distantly co‐occurring inter‐related entities constituted couple of errors. For example, in the sentence*CASE SUMMARY: A 65‐year‐old patient chronically treated with the selective serotonin reuptake inhibitor (SSRI) citalopram developed confusion, agitation, tachycardia, tremors, myoclonic jerks and unsteady gait, consistent with serotonin syndrome, following initiation of fentanyl, and all symptoms and signs resolved following discontinuation of fentanyl*(PMID:17381671); the relation between *confusion* and the last appearing drug name *fentanyl* was incorrectly classified. Case reports often contain frequencies at which potential adverse events were observed. For instance, *The toxic effects of methotrexate included elevated liver transaminases (3/4), nausea (2/4), abdominal pain (2/4), bone pain (2/4), mild neutropenia (1/4), and mild pruritus (1/4)*(PMID:433855); this sentence shows examples of relations where the system had difficulties in identification of correct relations. Potential adverse drug events are categorized according to their severity: serious suspected adverse drug reactions require immediate action by medical professionals. Manual investigation of the predicted results showed that the system was able to capture most of the serious potential adverse events. These findings demonstrate the potential of this approach to facilitate the identification of potential signals from case reports, of great interest for drug safety experts.

**Table 2:** Assessment of results of relation extraction

**Evaluation**	**Precision**	**Recall**	**F‐score**
Cross‐Validation	0.87	0.86	0.87
Final Test	0.86	0.89	0.87

### Impact of size of the training set on the performance

In order to study the impact of size of the training data on performance of classification, the ADE‐EXT‐TRAIN was decomposed into random subsets containing 10, 20, 50, 100, 200, 500, 1000, and 2000 documents. The JSRE was trained over these subsets independently in different rounds and evaluated by 10‐fold cross‐validation. Table 3 shows that already using 200 documents one could achieve performances over the 80% range. Whereby, to obtain a classifier with a standard deviation of 1%, one needs a substantially large training data.

**Table 3:** Impact of size of the training set on relation extraction

	**Precision**		**Recall**		**F‐score**	
**N**	**Mean**	**SD**	**Mean**	**SD**	**Mean**	**SD**
10	0.58	0.41	0.60	0.44	0.55	0.38
20	0.62	0.36	0.69	0.38	0.64	0.37
50	0.79	0.13	0.87	0.06	0.82	0.09
100	0.81	0.05	0.75	0.08	0.78	0.04
200	0.85	0.07	0.84	0.05	0.84	0.04
500	0.82	0.04	0.85	0.01	0.84	0.02
1000	0.83	0.02	0.87	0.02	0.86	0.01
2000	0.87	0.01	0.86	0.01	0.87	0.01

Impact of size of the training set on relation extraction was measured by independent cross‐validations over subsets of the ADE‐EXT‐TRAIN corpus. N indicates number of documents in the training set and SD indicates the standard deviation measured during the 10‐fold cross validation.

### Mapping the ADE annotation ontology to the ontology of adverse events

As clearly shown in Figure 2, both the ADE annotation ontology and OAE represent adverse drug reactions using formal ontological methods. In spite of this common goal, the two ontologies use different naming for the two core entities: a *Condition* in the ADE annotation ontology coincide with a *drug adverse event* in OAE; a *Drug* in the ADE annotation ontology coincide with a *drug‐administration* in OAE. The ADE ontology additionally introduce the entity *dosage*, not specified in OAE at the time of its development since OAE originally focused on vaccines for which dosing is not an essential medical concept. Both ADE and OAE model a causal relationship between *Condition* or *Adverse event* and *Drug* or *Medical intervention*, with the latter being the causal source. The only entity shared by the CLEF annotation ontology with OAE and ADE is the *Drug‐or‐device*, that coincide with a *Drug* or *Medical intervention*.

## Use case study: large scale relation extraction

An experiment was conducted in order to understand the real‐world use case scenarios for the extraction of potential adverse drug events from text. This was performed by applying the trained extraction tool to the whole MEDLINE and thereafter comparing them to the information present in drug leaflets present in the SIDER [21] database. Some of the automatically extracted potential adverse drug events, not present in SIDER, were manually investigated for their validity by comparison to the Medicines and Healthcare products Regulatory Agency (MHRA) drug label changes reported in 2009.

### Relation extraction from MEDLINE

MEDLINE articles published before 2009 were gathered to form a Medline‐2009 corpus. ProMiner was equipped with DrugBank and MedDRA dictionaries for tagging drugs and conditions occurring in sentences of Medline‐2009. A JSRE model trained over the ADE‐TRAIN‐EXT corpus was applied for classification of relations between drugs and conditions as *True* or *False* where a *True* relation indicates potential drug‐related adverse event. As a result of relation extraction, 165680 relations were extracted between 1611 drugs and 5079 adverse effects where drugs and adverse effects were normalized to DrugBank and MedDRA respectively.

### Adverse effect extraction from SIDER

Side Effect Resource (SIDER) is a database of adverse drug effects that links 888 drugs to 1450 adverse effects. It has been constructed manually from the summary of product leaflets of each drug. Drugs and their adverse effects were extracted from SIDER version 1.01 that contains drug leaflets published before 2009.

### MHRA drug label changes

In 2009, the MHRA proposed safety label updates for 26 drugs. These were of course not all the safety label updates that the MHRA identified in 2009, but those that MHRA decided to give particular visibility through their web site. These new adverse drug effects were manually extracted and they serve as a standard reference for validation of potential adverse drug events automatically extracted from Medline‐2009 using the JSRE trained method.

### Validation of large scale relation extraction

From the MHRA label change dataset, three drugs were arbitrarily chosen for deeper investigation. They are Rituximab, Efalizumab, and Natalizumab: three anti‐neoplastic and immunomodulatory monoclonal antibodies. For the three drugs of interest, potential adverse drug events were selected from the Medline‐2009 predictions and SIDER. Potential adverse drug events extracted from Medline‐2009 that are not reported in SIDER were manually checked against the label changes of MHRA.

Manual investigation of machine predicted potential adverse events showed that the system was able to capture valid potential adverse events from free‐text that were not yet reported in product leaflets (Table 4). These adverse effects were later updated on drug labels by the UK regulatory authorities. This instance provides a good example for how the developed framework can help in capturing potential adverse drug events from literature and therefore support pharmacovigilance.

**Table 4:** Potential adverse drug events extracted from MEDLINE not reported in drug leaflets until 2009 and later introduced in package leaflets

**Drug**	**Adverse effect**
Rituximab	Progressive multifocal leukoencephalopathy
Efalizumab	Progressive multifocal leukoencephalopathy
Natalizumab	Hypersensitivity

## Conclusions

This work reports on the adaptation of a machine learning‐based JSRE system for the identification and extraction of potential adverse events of drugs in scientific case reports. A methodology has been discussed to enrich a sparsely annotated corpus and its subsequent use to build classification models. Evaluation of the system’s performance showed promising results. A use‐case study performed on relation extraction from large scale literature showed the system’s ability to capture valid, under‐reported, and novel potential adverse events not yet present in product leaflets.

The performance of the system can be improved in several ways. In the current experiments, only the default features acceptable by JSRE were used. Optimization of feature representation to include additional features for instance from syntactic sentence parse trees may further improve the results. Development of additional strategies like post‐processing to classify relations with missing contextual descriptions can help to recover more relations. Furthermore, extension of handling inter‐sentence relations needs to be considered in order to further increase coverage.

The reported experimental results denote the research status on identification from text of potential adverse drug events. There are several strategies that are being followed. The authors plan to benchmark the performances of several named entity taggers against the ADE corpus for the identification of drugs and conditions mentions in text. The current experiments have been performed on the ADE corpus, since that was the only one available when this work was done, however while writing this report a new corpus has been published, namely the EU‐ADR corpus [22]. It will be interesting to see if the performance of JSRE on the ADE corpus will be different compared to the EU‐ADR corpus.

Similarly, benchmarking results of public and commercial relation extraction systems will be performed [23] and the practical impact of the information extracted from text on predicting drug label changes will be studied in detail.

The use of ontologies for driving information extraction has been reported [24,25]. We plan to explore the use of various available tools (e.g. ODIE, OBCIE,semantixs) using the OAE ontology and compare the performance of the ontology driven / based methods for information extraction against the method presented here.

The current work has demonstrated promising results, it has the potential to reduce the manual reading time, improve the quality of the signal detection process, and therefore positively contribute to safer use of drugs to the benefit of patients and society. We speculate that this work could also pave the road to pharmacovigilance applications on social media and multimedia sources too.

## Competing interests

LT is employee of Merck KGaA. AM‐R was founded by Merck KGaA. HG has no conflicts of interests to declare.

## References

[B1] Hauben M Bate A Decision support methods for the detection of adverse events in post‐marketing data Drug Discov Today 2009 14 7‐8 343 357 10.1016/j.drudis.2008.12.012 19187799

[B2] Vandenbroucke JP In defense of case reports and case series Ann Intern Med 2001 134 4 330 334 11182844 10.7326/0003-4819-134-4-200102200-00017

[B3] Wang X Hripcsak G Markatou M Friedman C Active computerized pharmacovigilance using natural language processing, statistics, and electronic health records: a feasibility study J Am Med Inform Assoc 2009 16 3 328 337 10.1197/jamia.M3028 19261932 PMC2732239

[B4] Leaman R Wojtulewicz L Sullivan R Skariah A Yang J Gonzalez G Dina Demner‐Fushman K, Cohen Bretonnel, Ananiadou Sophia, Pestian John, Tsujii Jun’ichi, Webber Bonnie Towards internet‐age pharmacovigilance: extracting adverse drug reactions from user posts to health‐related social networks Proceedings of the 2010 Workshop on Biomedical Natural Language Processing 2010: Uppsala, Sweden 117 125 http://delivery.acm.org/10.1145/1870000/1869976/p117–leaman.pdf

[B5] Gurulingappa H Fluck J Hofmann‐Apitius M Toldo L Rangwala H, Tagarelli A, Wale N, Karypis G Identification of Adverse Drug Event Assertive Sentences in Medical Case Reports First International Workshop on Knowledge Discovery and Health Care Management (KD‐HCM), European Conference on Machine Learning and Principles and Practice of Knowledge Discovery in Databases (ECML PKDD) 2011 Athens, Greece: 16‐27 16‐27 http://www.cs.gmu.edu/hrangwal/kd–hcm/proc/KDHCM11_procs.pdf

[B6] Henegar C Bousquet C Lillo‐Le Louet A Degoulet P Jaulent MC Building an ontology of adverse drug reactions for automated signal generation in pharmacovigilance Comput Biol Med 2006 36 748 767 10.1016/j.compbiomed.2005.04.009 16185681

[B7] Delamarre D Lillo‐Le Louët A Guillot L Jamet A Sadou E Ouazine T Burgun A Jaulent MC Documentation in pharmacovigilance: using an ontology to extend and normalize Pubmed queries Stud Health Technol Inform 2010 160 Pt 1 518 522 20841741

[B8] Aramaki E Miura Y Tonoike M Ohkuma T Masuichi H Waki K Ohe K Safran C Extraction of adverse drug effects from clinical records MEDINFO 2010 ‐ Proceedings of the 13th World Congress on Medical informatics, Series: Studies Health Technology Informatics, Volume 160 2010 Cape Town, South Africa: IOS Press 739‐743 739‐743–1–60750–588–4–739 10.3233/978 20841784

[B9] Benton A Ungar L Hill S Hennessy S Mao J Chung A Leonard C Holmes J Identifying potential adverse effects using the web: A new approach to medical hypothesis generation J Biomed Informatics 2011 44 989 996 10.1016/j.jbi.2011.07.005 PMC4404640 21820083

[B10] Roberts A Gaizauskas R Hepple M Demetriou G Guo Y Roberts I Setzer A Building a semantically annotated corpus of clinical texts J Biomed Informatics 2009 42 950 966 10.1016/j.jbi.2008.12.013 19535011

[B11] Gurulingappa H Mateen‐Rajput A Roberts A Fluck J Hofmann‐Apitius M Toldo L Development of a Benchmark Corpus to Support the Automatic Extraction of Drug‐related Adverse Effects from Medical Case Reports J Biomed Informatics 2012 45 885 892 10.1016/j.jbi.2012.04.008 22554702

[B12] Hanisch D Fundel K Mevissen HT Zimmer R Fluck J ProMiner: rule‐based protein and gene entity recognition BMC Bioinformatics 2005 6 Suppl 1:S14 [–2105–6–S1–S14] 10.1186/1471 PMC1869006 15960826

[B13] Knox C Law V Jewison T Liu P Ly S Frolkis A Pon A Banco K Mak C Neveu V Djoumbou Y Eisner R Guo AC Wishart DS DrugBank 3.0: a comprehensive resource for ’omics’ research on drugs Nucleic Acids Res 2011 39 Database issue D1035—D1041 10.1093/nar/gkq1126 21059682 PMC3013709

[B14] Merrill GH The MedDRA paradox Proceedings of the AMIA 2008 Annual Symposium 2008 Washington, DC, USA: 470 474 http://www.ncbi.nlm.nih.gov/pmc/articles/PMC2655972/pdf/amia–0470–s2008.pdf PMC2655972 18998828

[B15] Giuliano C Lavelli A Pighin D Romano L Richard W, Lluís M, Agirre E, Lluís M, Richard W FBK‐IRST: Kernel Methods for Semantic Relation Extraction Proceedings of the Fourth International Workshop on Semantic Evaluations 2007 Prague, Czech Republic: 141‐144 141‐144 http://aclweb.org/anthology–new/S/S07/S07–1000.pdf

[B16] Burges C A Tutorial on Support Vector Machines for Pattern Recognition Data Mining and Knowledge Discovery 1998 2 121‐167

[B17] Tikk D Thomas P Palaga P Hakenberg J Leser U A comprehensive benchmark of kernel methods to extract protein‐protein interactions from literature PLoS Comput Biol 2010 6 e1000837 10.1371/journal.pcbi.1000837 20617200 PMC2895635

[B18] Roberts A Gaizauskas R Hepple M Demetriou G Guo Y Roberts I Setzer A The CLEF corpus: semantic annotation of clinical text Proceedings of the AMIA Symposium 2007 Chicago, IL, USA: 625 629 http://www.ncbi.nlm.nih.gov/pmc/articles/PMC2655900/pdf/amia–0625–s2007.pdf PMC2655900 18693911

[B19] Ogren P Moore Robert C, Bilmes Jeff, Chu‐Carroll Jennife, Sanderson Mark Knowtator: a Protégé plug‐in for annotated corpus construction Proceedings of the 2006 conference of the North American chapter of the association for computational linguistics on human language technology 2006 New York, NY, USA: 273 275 http://aclweb.org/anthology–new/N/N06/N06–4006.pdf

[B20] Yongqun H Zuoshuang X Sarntivijai S Toldo L Ceusters W Courtot M, Goldfain A, Yongqun He O, Ruttenberg A AEO: A Realism‐Based Biomedical Ontology for the Representation of Adverse Events “Representing Adverse Events” at the International Conference on Biomedical Ontology 2011 NY, USA: Buffalo http://icbo.buffalo.edu/2011/workshop/adverse–events/docs/papers/HeAEICBO2011_ submission.pdf

[B21] Kuhn M Campillos M Letunic I Jensen LJ Bork P A side effect resource to capture phenotypic effects of drugs Mol Syst Biol 2010 6 343 10.1038/msb.2009.98 20087340 PMC2824526

[B22] van Mulligen E Fourrier‐Reglat A Gurwitz D Molokhia M Nieto A Trifiro G Kors J Furlong L The EU‐ADR Corpus: Annotated Drugs, Diseases, Targets, and their Relationships J Biomed Informatics 2012 45 879 884 10.1016/j.jbi.2012.04.004 22554700

[B23] Toldo L Gurulingappa H Mateen‐Rajput A Kors J Suri S Tayrouz Y Impact of Automatic Detection of Adverse Events on Prediction of Drug Label Changes J Pharmacoepidemiology and Drug Saf 2012 [Submitted]

[B24] Wimalasuriya D Dou D Ontology‐based information extraction: an introduction and a survey of current approaches J Information Sci 2010 36 306 323 10.1177/0165551509360123

[B25] Pandit S Honavar V Pierre M Ontology‐guided extraction of complex nested relationships22nd IEEE International Conference on tools with artificial intelligence (ICTAI) 2010 France: Arras 173 178 http://dx.doi.org/10.1109/ICTAI.2010.98

